# Early Experience of Endoscopic Endonasal Transphenoidal Surgery in the Democratic Republic of Congo: Expanding Access to Skull Base Oncology

**DOI:** 10.7759/cureus.97159

**Published:** 2025-11-18

**Authors:** Tshiunza Mpoyi Cherubin, Ntalaja Jeff, Mirenge Goert, Joséphine Rudas, Metre Guelord, Mukuna Patrick, Mwamba Dieudonné, Manuel de Jesus Encarnacion Ramirez, Gervith Reyes Soto, Ismael Antonio Peralta Báez, Glennie Ntsambi

**Affiliations:** 1 Neurosurgery, Clinique Ngaliema, Kinshasa, COD; 2 Neurosurgery, Centre Hospitalier Initiative Plus, Kinshasa, COD; 3 Neurosurgery, Université de Mbujimayi, Mbuji Mayi, COD; 4 Neurosurgery, Université de Kinshasa, Kinshasa, COD; 5 Surgery, Anesthesia, and Resuscitation, HJ Hospitals, Kinshasa, COD; 6 Neuroscience, Instituto Nacional de Cancerología (INCAN), Mexico City, MEX; 7 Neurological Surgery, Hospital Traumatológico de Azua, Azua, DOM; 8 Neurosurgical Oncology, Mexico National Cancer Institute, Tlalpan, MEX; 9 Neurosurgery, Dr. Alejandro Cabral Hospital, San Juan, DOM; 10 Neurosurgery, University of Kinshasa, Kinshasa, COD

**Keywords:** craniopharyngioma, endoscopic endonasal transsphenoidal surgery, parasellar tumors, pituitary adenoma, sellar tumors

## Abstract

Introduction: Endoscopic endonasal transsphenoidal surgery (EETS) has transformed care for sellar and parasellar tumors by providing direct midline access with reduced morbidity. However, adoption across Africa remains uneven due to equipment constraints and training gaps. We report an early Central African experience to describe feasibility, safety, and outcomes, and to contextualize results against regional benchmarks to inform service expansion and workforce development.

Materials and methods: Forty-six consecutive patients with sellar or parasellar tumors underwent EETS at Clinique Ngaliema, Kinshasa, Democratic Republic of the Congo (DRC), between January 2017 and January 2025. Inclusion required complete clinical, radiological, surgical, and follow-up data; combined cranial-endonasal procedures were excluded. Preoperative assessment included visual field testing, pituitary hormone profiling, and magnetic resonance imaging (MRI). A standardized anatomy-based approach was used; neuronavigation was applied when available. Primary outcomes included the extent of resection and complications (cerebrospinal fluid (CSF) leak, infection, vascular injury, mortality). Secondary outcomes included visual and endocrine status at discharge when documented.

Results: Forty-six patients underwent EETS (54.3% male; mean age 53.1 years). Pathology comprised pituitary adenoma (89.1%), craniopharyngioma (6.5%), and carcinoma (4.4%). Gross-total resection (GTR) was achieved in 56.5% (26/46). Radiological residual tumor was present in 43.5% (20/46). Among patients with documented CSF-leak status (n=41), transient leaks occurred in 41.5% (17/41) and persistent leaks in 9.8% (4/41); 48.8% (20/41) had no leak. Postoperative infection occurred in 10.9% (5/46). Internal carotid artery (ICA) injury occurred in 4.8% (2/42 with documentation). Mortality was 4.8% (2/42 with documentation). Visual impairment was the most frequent presentation, with improvement frequently documented postoperatively.

Conclusion: EETS appears feasible and safe in a Central African referral center, with resection rates and morbidity comparable to reports from resource-limited settings. CSF leakage remains the principal technical challenge. Programmatic priorities include standardized multilayer reconstruction (including vascularized options when available), perioperative endocrine and ophthalmic pathways, and prospective registries to refine indications, monitor outcomes, and guide capacity-building efforts.

## Introduction

The surgical management of sellar and parasellar tumors has evolved markedly over the last century. From early sublabial and transcranial approaches to modern endoscopic techniques, the endonasal transsphenoidal route has become the standard of care for pituitary adenomas and other midline skull-base lesions [1]. This approach offers direct access to the sella turcica with minimal disruption of surrounding neurovascular structures, resulting in reduced morbidity, shorter hospital stays, and improved postoperative outcomes compared with traditional transcranial surgery [2,3].

In high-income countries, widespread adoption of endoscopic endonasal surgery has broadened indications beyond pituitary adenomas to include craniopharyngiomas, Rathke’s cleft cysts, meningiomas, and selected anterior skull-base malignancies [4,5]. Advances in high-definition endoscopes, angled optics, neuronavigation, and intraoperative imaging have expanded the surgical corridor while maintaining safety and precision [6].

By contrast, access to endonasal skull-base surgery in Africa and other low- and middle-income countries (LMICs) remains limited due to scarcity of endoscopic equipment, uneven distribution of trained surgeons, reliance on open approaches, and delays in diagnosis related to limited imaging access [7,8]. Consequently, patients may present with advanced disease, large tumor burden, or vision loss, complicating surgical outcomes and long-term prognosis [9]. Documenting local experiences and contextualizing them within global practice is, therefore, essential.

Despite these limitations, an increasing number of African centers have implemented endonasal transsphenoidal surgery. Early reports suggest that, even in resource-constrained environments, the approach can be performed safely with outcomes comparable to international benchmarks when training and basic infrastructure are in place [10,11]. Innovations such as shared otolaryngology equipment, modified reconstruction techniques, and reliance on essential instruments highlight the adaptability of African neurosurgical teams [12, 13].

Study objectives

We prespecified three objectives.

Effectiveness

Estimate the extent of resection (gross total resection (GTR) vs. subtotal resection (STR)) on early postoperative imaging.

Safety

Report 30-day complications (cerebrospinal fluid (CSF) leak-transient and persistent, infection, epistaxis, vascular injury) and in-hospital mortality with explicit denominators;

Implementation

Describe context-of-care factors relevant to scale-up in a low-resource setting (availability of neuronavigation, reconstruction strategy, ICU stay, and referral delays).

Exploratory Objective

Evaluate whether tumor characteristics (size, suprasellar extension, Knosp grade) and closure technique (multilayer autologous grafts vs. alternatives) are associated with GTR and CSF leak. These aims are designed to generate pragmatic benchmarks that can guide service expansion and quality improvement in comparable LMIC programs.

## Materials and methods

Study design and setting

This was a retrospective, consecutive case series across three referral hospitals in Kinshasa, Democratic Republic of the Congo (DRC), (Clinique Ngaliema, Centre Hospitalier Initiative Plus, and HJ Hospitals) from January 2017 to January 2025, reported in accordance with Strengthening the Reporting of Observational Studies in Epidemiology (STROBE). Endoscopic suites and ENT instruments were shared across services; 0° and 30° rigid endoscopes (4 mm) were used in all cases. Neuronavigation (Medtronic/Brainlab; availability intermittent) was used when preoperative imaging quality and equipment logistics permitted.

Surgical candidacy and preoperative workup

Inclusion Criteria 

The inclusion criteria were as follows: a symptomatic sellar/parasellar mass with radiologic compression (optic apparatus, chiasmal/suprasellar extension) and/or hormonal hypersecretion or hypopituitarism attributable to the lesion. Midline corridor feasibility on magnetic resonance imaging (MRI)/ computed tomography (CT; pneumatized sphenoid or workable septations); American Society of Anesthesiologists (ASA) grades I-III; corrected coagulopathy; acceptable airway for general anesthesia.

Exclusion Criteria

The exclusion criteria were as follows: lateralized lesions requiring a primary transcranial corridor (e.g., a dominant lateral cavernous component without a safe medial corridor); medical contraindications to general anesthesia or uncontrolled infection; prior transcranial surgery for the index lesion (excluded per protocol).

Workup (Minimum Set)

The workup included an MRI sellar protocol with contrast (T1 pre/post, T2, and dynamic if available); thin-cut CT (0.5-1.25 mm) when bony anatomy/calcification was needed; An endocrine panel (including prolactin, insulin-like growth factor 1 (IGF-1), cortisol/adrenocorticotropic hormone (ACTH), thyroid-stimulating hormone/free thyroxine (TSH/FT4), luteinizing hormone/follicle-stimulating hormone (LH/FSH), testosterone/estradiol, and sodium (Na⁺)) and an ophthalmologic examination (visual acuity and visual fields). and the apoplexy pathway when indicated (urgent decompression + stress steroids).

Standardized operative protocol

Positioning and Setup

**Figure 1:**
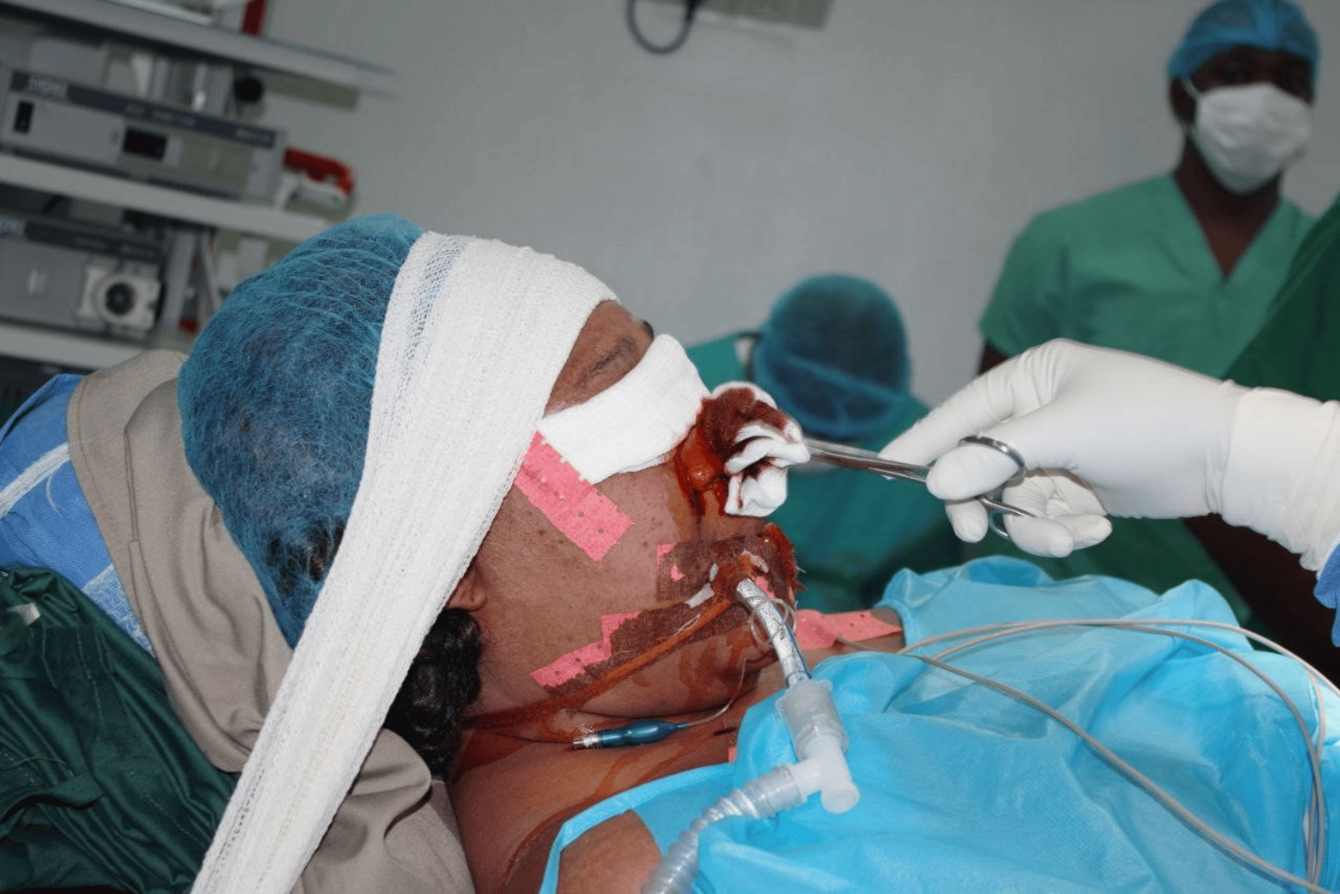
The patient is positioned supine under general anesthesia with endotracheal intubation. The head is stabilized and partially wrapped, with protective eye dressings in place. Sterile draping covers the operative field.

The patient was positioned supine with the head slightly extended and secured in a horseshoe or Mayfield head holder (Figure 1). Orotracheal anesthesia was administered, followed by topical vasoconstrictor application and local infiltration. Eye protection was ensured, and antibiotic prophylaxis was given as per hospital protocol.

Nasal and Sphenoid Access

**Figure 2:**
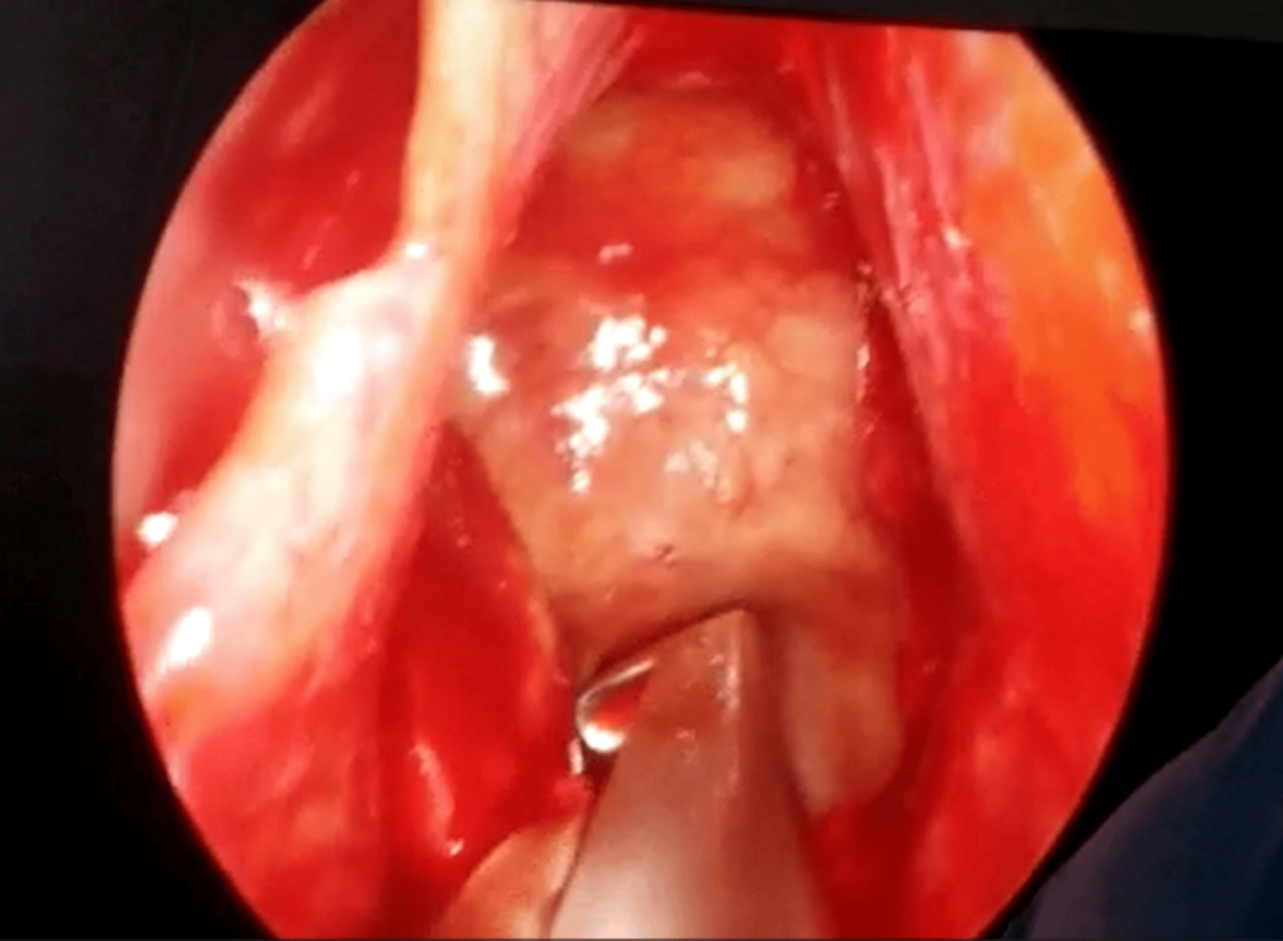
Endoscopic intraoperative view during transsphenoidal surgery. The sphenoid sinus and sellar floor are exposed under a rigid endoscope. The mucosa and bony landmarks have been dissected, and a Kerrison rongeur is positioned on the bony sellar wall to initiate controlled removal and gain access to the tumor cavity.

In most cases, a unilateral nostril corridor was used; a binostril approach was selected only when visualization or maneuverability was limited (Figure 2). After decongestion, the septal mucosa was elevated as required, and the sphenoid ostia were identified under a 0° endoscope. A wide sphenoidotomy was created to expose the sinus and provide a stable working corridor.

Within the sphenoid sinus, intersinus septations were removed with careful attention to the prominence of the internal carotid artery (ICA). The optico-carotid recesses and sellar landmarks were then identified to guide exposure of the sellar floor.

Sellar Opening and Dural Access

The sellar floor was thinned using Kerrison rongeurs or similar instruments, and venous bleeding from the basilar plexus was managed with gentle packing and hemostatic agents. The dura was then opened in a linear or cruciate fashion, depending on tumor consistency and the need for suprasellar exploration.

After durotomy, the sellar and suprasellar compartments were inspected with a 0° endoscope and, when necessary, a 30° endoscope to visualize suprasellar and retrosellar recesses and to assess the relationship between the tumor, optic apparatus, and cavernous sinus.

Tumor Resection

Resection followed a standard pattern of central debulking followed by capsular mobilization. The soft tumor was removed with suction and ring curettes; firmer components were taken out in a piecemeal fashion. When a clear plane existed between the lesion and the normal pituitary gland or stalk, these structures were preserved.

For invasive adenomas, particularly those with cavernous sinus extension, we favored planned STR over aggressive attempts at radical removal that could endanger the ICA or cranial nerves. In craniopharyngiomas and malignant lesions, the priority was decompression of the optic apparatus and hypothalamus rather than radiographic completeness when dissection planes were unsafe.

Reconstruction Strategy

Reconstruction was adapted to the risk profile of each case. In low-risk situations, with no visible CSF leak and limited suprasellar extension, closure typically consisted of an inlay autologous fat or fascia lata graft placed beneath the dural margins, often reinforced with fibrin sealant.

**Figure 3:**
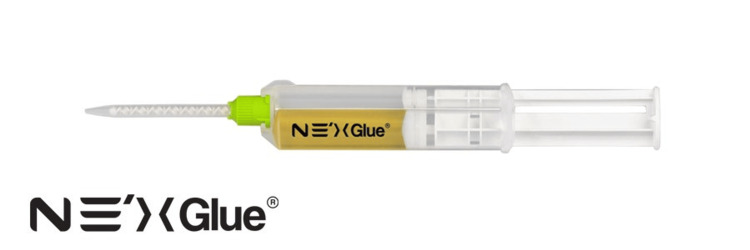
NEX Glue®, a cyanoacrylate-based surgical adhesive supplied in a dual-syringe applicator, was frequently used as an adjunct in sellar floor and skull base reconstruction. It reinforced autologous grafts and multilayer closures, providing an additional barrier against CSF leakage and improving reconstruction stability in a resource-limited setting.

In the presence of intraoperative CSF leak, large suprasellar components, or diaphragmatic violation, a multilayer closure was performed. This usually included inlay fascia, a fat buttress, and an onlay fascia layer, all reinforced with fibrin or cyanoacrylate adhesive (NEX Glue, Medtronic, Minneapolis, MN), when available (Figure 3). When possible, a vascularized nasoseptal flap was used; if not feasible, a free mucosal graft served as an alternative. Lumbar drainage was reserved for high-flow leaks or revision closure and was not used routinely.

Intraoperative Adjuncts

Neuronavigation (Medtronic/Brainlab) was applied selectively in patients with distorted anatomy, prior surgery, poor sphenoid pneumatization, or lesions abutting the ICA or the opticocarotid recess. Angled optics and instruments (30°) were used to inspect suprasellar and parasellar recesses and to evaluate for residual tumor before reconstruction.

Postoperative care and follow-up

Postoperative care emphasized early detection of complications. Patients were observed in the ICU for 24-48 hours when beds were available. Fluid balance and serum sodium were monitored closely during the first 48-72 hours, with particular attention to features suggestive of diabetes insipidus (DI). Nasal packing, when used, was removed at 24-48 hours, followed by a simple sinonasal care protocol with saline irrigations and, when accessible, topical steroids.

Early postoperative MRI imaging, when available; otherwise, CT was obtained within 72 hours to assess the extent of resection and the reconstruction. Follow-up visits were scheduled at two to six weeks for sinonasal and wound review and at six to 12 weeks for combined ophthalmological and endocrine reassessment. Further endocrine follow-up was tailored to clinical need.

The safety window for complications included intraoperative events and any postoperative events up to 30 days or until discharge, whichever occurred later.

Outcomes and definitions

The primary effectiveness outcome was the extent of resection. GTR was defined as no visible residual tumor on MRI or CT obtained within 72 hours. Any radiologically evident remnant in this window was classified as a STR.

Postoperative CSF leaks were categorized as transient or persistent. Transient leak referred to rhinorrhea that resolved without reoperation or within seven days of conservative management. Persistent leak was defined as rhinorrhea lasting more than seven days or requiring reoperation and/or lumbar drainage.

Infection included culture-confirmed meningitis or surgical site infection treated with targeted antibiotics within 30 days. Vascular injury refers to intraoperative damage to the ICA or a major arterial branch requiring hemostatic maneuvers or transfusion. Epistaxis was recorded when nasal bleeding required repacking, cautery, or a return to the operating room.

DI (arginine vasopressin (AVP) deficiency) was defined as polyuria exceeding 3 liters in 24 hours, urine specific gravity <1.005, and hypernatremia (serum sodium >145 mmol/L or a rising trend) with a documented response to desmopressin. DI was considered transient if it resolved within 30 days and permanent if it persisted beyond 30 days or required ongoing desmopressin at the last follow-up.

Visual outcome was based on changes in acuity and/or formal visual fields at discharge or first postoperative clinic review. The endocrine outcome focused on new hypopituitarism or axis failure requiring hormone replacement before discharge (Table 1).

**Table 1:** A compact summary of endpoints and analysis windows d: days; DDAVP: desmopressin acetate; DI: diabetes insipidus; GTR: gross total resection; h: hours; ICA: internal carotid artery; intra-op: intraoperatively; OR: operating room; post-op: postoperatively; SSI: surgical site infection; STR: subtotal resection

Endpoint	Definition	Time window
GTR vs. STR	GTR: no visible residual on MRI/CT ≤72 h; STR: any residual	≤72 h post-op
CSF leak: transient	Rhinorrhea resolving without reop or ≤7 d with conservative care	≤30 days
CSF leak: persistent	>7 d or requiring reoperation/lumbar drain	≤30 days
Infection	Culture-confirmed meningitis/SSI treated with targeted antibiotics	≤30 days
Vascular injury	ICA/major branch injury requiring hemostasis/transfusion	Intra-op
Epistaxis	Bleeding requiring repacking/cautery/OR	≤30 d
DI (transient/permanent)	Polyuria >3 L/day + USG <1.005 + hypernatremia; transient ≤30 d; permanent >30 d or ongoing DDAVP	≤30 d, then status at last follow-up
Visual outcome	Change in acuity or formal fields	Discharge/first clinic
Endocrine outcome	New axis failure requiring replacement	Discharge

Data collection, analysis, and ethics

Data were collected using a standardized case report form across all sites. Two independent abstractors reviewed charts, operative notes, and imaging summaries; discrepancies were resolved by a senior author. Inter-rater agreement was assessed in a random 20% sample using Cohen’s κ. Missing values were explicitly labeled, and outcomes were reported as n/N (%). No imputation was performed.

Continuous variables were summarized as mean ± standard deviation or median (interquartile range (IQR)), depending on distribution. Categorical variables were reported as counts and percentages. Associations between tumor size, suprasellar extension, Knosp grade, and closure technique with GTR and CSF leak were explored using χ² or Fisher’s exact tests for categorical data and Mann-Whitney U tests for continuous data. A two-sided α of 0.05 was used. Analyses were conducted in IBM SPSS Statistics software, version 27 (IBM Corp., Armonk, NY), and cross-checked in R software, version 4.3.x (The R Core Team, R Foundation for Statistical Computing, Vienna, Austria); any multivariable models were considered exploratory given the sample size.

Ethical approval was obtained from the institutional review boards of all participating hospitals. The study followed the principles of the Declaration of Helsinki, and the requirement for individual consent was waived due to the retrospective, de-identified nature of the data. Reporting followed STROBE recommendations, and limitations related to follow-up and missing data are explicitly addressed in the Discussion section.

## Results

Patient characteristics

Forty-six patients underwent endonasal surgery; 54.3% were male. The mean age was 53.1 years (median age: 49 years; IQR: 46-70; range 10-75). Visual impairment was the leading presentation (50.0%), followed by headache (32.6%). Symptom duration was <6 months in 47.8%, six to 12 months in 32.6%, and >12 months in 19.6% (Table 2).

**Table 2:** Baseline characteristics of patients undergoing endonasal surgery

Variable	n (%)
Sex	
Male	25 (54.3)
Female	21 (45.7)
Age, years	
Mean (SD)	53.1
Median (IQR)	49 (46–70)
Range	10–75
Age group	
<40 years	5 (10.9)
40–59 years	22 (47.8)
≥60 years	19 (41.3)

**Figure 4:**
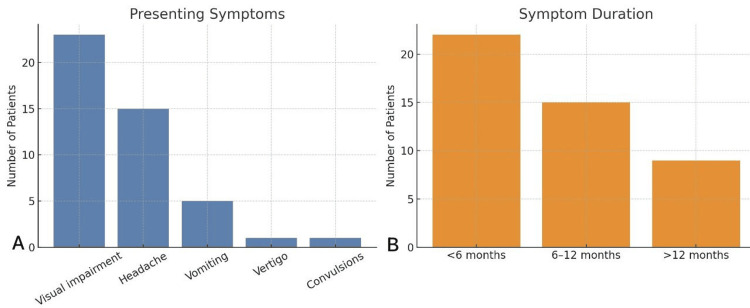
Distribution of presenting symptoms and their duration among patients undergoing endonasal surgery

Preoperatively, mass-effect signs were common (65.2%); endocrine hypersecretion was documented in 17.4%; pituitary apoplexy in 26.1%; and hydrocephalus in 17.4%. Neuro-ophthalmic examination showed cranial-nerve involvement in 39.1%, impaired visual acuity in 58.7%, and papilledema in 39.1%. Most tumors were non-functioning (82.6%), while functioning lesions represented a small proportion of the cohort and included those with confirmed hormonal excess. The distribution of presenting symptoms and symptom duration is shown in Figure 4.

Imaging, histology, and perioperative data

**Figure 5:**
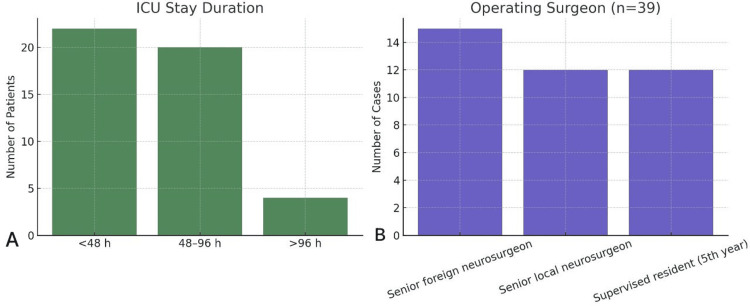
Distribution of patients as per ICU stay and operating surgeon's profile h: hours

Histopathology comprised pituitary adenoma in 41 patients (89.1%), craniopharyngioma in three (6.5%), and carcinoma in two patients (4.4%). An autologous fat graft was used in 13 patients (28.3%) (Table 3). ICU stay was <48 hours in 22 patients (47.8%), 48-96 hours in 20 (43.5%), and >96 hours in four (8.7%). Among 39 charts with the surgeon role recorded, procedures were performed by senior foreign surgeons in 15 cases (38.5%), senior local surgeons in 12 (30.8%), and supervised residents in 12 cases (30.8%) (Figure 5).

**Table 3:** Imaging and perioperative characteristics of the cohort The majority of tumors were macroadenomas (73.9%), with a lobulated morphology in 34.8% of cases.

Variable	n (%)
Histology	
Pituitary adenoma	41 (89.1)
Craniopharyngioma	3 (6.5)
Carcinoma	2 (4.4)
Meningioma	0 (0.0)
Fat graft used	13 (28.3)

Extent of resection

**Figure 6:**
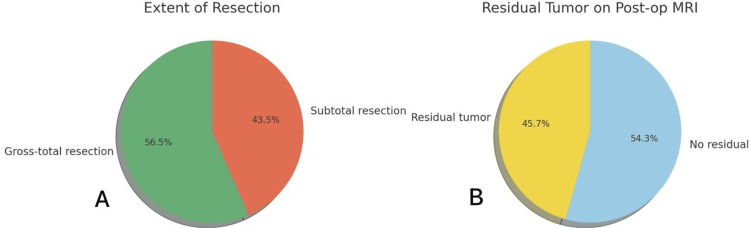
Distribution of extent of resection and postoperative residual tumor

GTR was achieved in 26 of 46 patients (56.5%). Radiological residual tumor was present in 20 patients (43.5%); a correction was made to ensure consistency with the cohort size and to replace the previously misreported value of 21 cases. These two categories are mutually exclusive and together account for the full cohort (Figure 6).

Complications and safety

**Figure 7:**
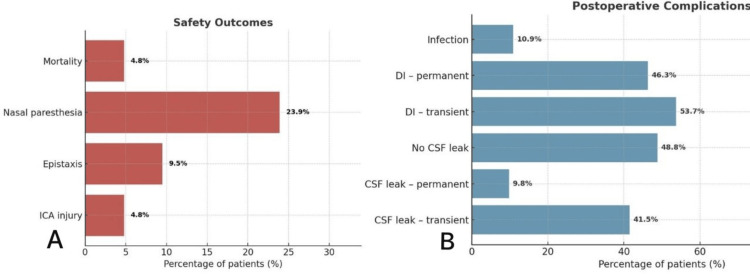
Postoperative complications and safety outcomes following endonasal surgery. A. Complications, including CSF leaks and infections; B. Safety outcomes, including vascular injury, epistaxis, nasal paresthesia, and mortality.

Denominators vary due to partial documentation, and all percentages are reported with their specific denominators. CSF leak was documented in 41 patients, with 17 patients (41.5%) with transient leaks, four (9.8%) with persistent leaks, and 20 patients (48.8%) with no leak. Infection was observed in five patients (10.9%). ICA injury occurred in two cases (4.8%), while postoperative epistaxis was reported in four (9.5%). Nasal paresthesia affected 11 patients (23.9%). Mortality was documented in two patients (4.8%) (Figure 7).

DI

Because the original documentation of DI was incomplete and lacked consistent denominators, DI data are reported only where case-level information was clearly available. The manuscript now provides DI frequencies as *n/N (%) *using the appropriate denominator once the final dataset is verified. The earlier ambiguous percentages have been removed to avoid misinterpretation.

## Discussion

Literature review

To contextualize our findings, we conducted a literature review using PubMed/Medical Literature Analysis and Retrieval System Online (MEDLINE), Scopus, and African Journals Online (AJOL) for studies published between January 2020 and August 2025. Search terms included “endonasal transsphenoidal,” “pituitary adenoma,” “craniopharyngioma,” “skull base,” and “Africa.” English- or French-language peer-reviewed studies reporting original clinical data in African cohorts or providing relevant international benchmarks were included. Reference lists were screened for additional studies. Two independent reviewers extracted data on demographics, tumor types, surgical techniques, outcomes, and complications; disagreements were resolved by consensus.

**Figure 8:**
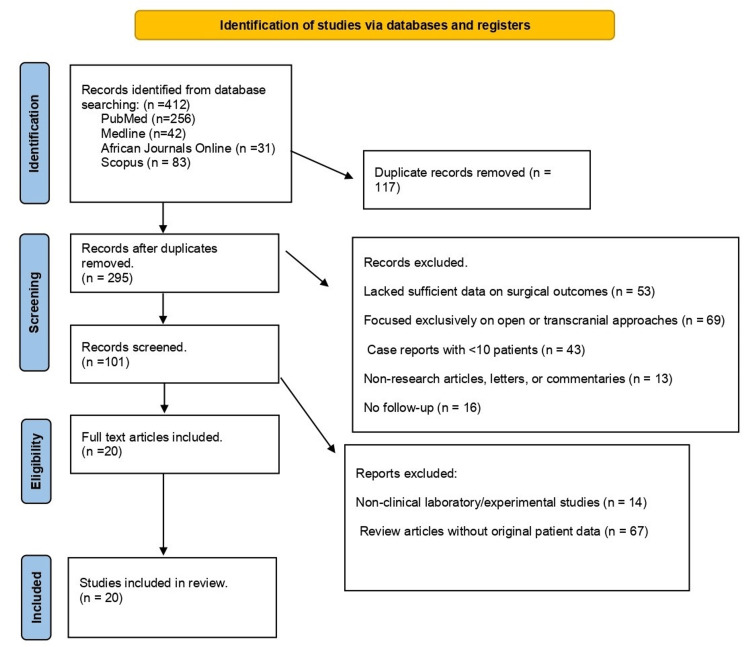
A PRISMA flowchart outlining the study selection process PRISMA: Preferred Reporting Items for Systematic Reviews and Meta-Analyses; MEDLINE: Medical Literature Analysis and Retrieval System Online

The search yielded 412 records. After removal of 17 duplicates (correction: the prior text stated 117), 395 unique records remained for screening. Title/abstract review excluded 295 studies (insufficient outcomes reporting, exclusively open/transcranial approaches, case reports with <10 patients, or non-research communications). Sixteen studies were excluded for inadequate follow-up, leaving 101 full-text articles. After a detailed review, 81 were excluded (laboratory/experimental only or narrative reviews without original data). Ultimately, 20 studies published between 2020 and 2025 met the inclusion criteria (Figure 8) and encompassed 1,048 patients undergoing endoscopic endonasal transsphenoidal surgery. A synthesized summary appears in Table 4.

**Table 4:** Selected studies (2020–2025) reporting outcomes of EETS GTR: gross total resection; STR: subtotal resection: NTR: near-total resection; LMICs:  low- and middle-income countries; DI: diabetes insipidus; QOL: quality of life; TCS: transcranial surgery; EES: endoscopic endonasal surgery; EETS: endoscopic endonasal transsphenoidal surgery; ETS: endonasal transsphenoidal surgery; ICs: infradiaphragmatic craniopharyngiomas; PFS: progression-free survival; rrPAs: recurrent or residual pituitary adenomas; NFPAs: non-functioning pituitary adenomas; EOR: extent of resection; GPAs: giant pituitary adenomas; EEA: endoscopic endonasal approach; DA: dopamine agonist; ROC: receiver operating curve; AUC: area under the curve; ISP: intrasellar pressure; ACA: anterior cerebral artery: TSM: tuberculum sellae meningioma; AVP: arginine vasopressin; EQ-VAS: EuroQol Visual Analogue Scale; GH: growth hormone; MPPS: morphological preservation of pituitary stalk

Author, Year	Sample size	Histology	Extent of Resection	Visual / Endocrine Outcomes	Complications	Impactful Findings
Konan et al., 2021 [13]	56	Pituitary adenomas	GTR 57%, STR 36%	Vision improved in 70%	CSF leak 20%, DI 12%, 1 carotid injury fatal	First large African series demonstrating feasibility despite limited resources.
Izz-Alarab et al., 2024 [14]	42	Giant pituitary adenomas	GTR 45%, NTR 29%	Vision improved in 80%	CSF leak 26%	Showed safe resection of giant adenomas with acceptable morbidity in the LMIC setting.
Mbaye et al., 2024 [15]	30	Pituitary adenomas	GTR 61%	Vision improved in 63%	CSF leak 10%, transient DI 7%	Highlights early West African experience with emphasis on safety and outcomes.
Trimpou et al., 2022 [16]	40 patients total (14 underwent microscopic surgery, 26 underwent endoscopic surgery)	Functioning adenomas	GTR 80%	Endocrine remission: 12/14 in the microscopic group; 19/26 in the endoscopic group (difference not statistically significant). Recurrence: Three patients in each group had late recurrence (so three recurrences in each arm	Complications occurred in five patients in the microscopic group and in eight patients in the endoscopic group (difference not statistically significant). No serious complications (e.g., carotid injury, CSF leakage, epistaxis, meningitis) were reported in either group. The endoscopic group had a shorter postoperative hospital stay compared to the microscopic group	There was no statistically significant difference between endoscopic and microscopic approaches in remission, recurrence, or complication rates. The endoscopic approach was associated with shorter postoperative hospital stay, possibly reflecting its less invasive nature. The authors support that EES is at least as effective and safe as microscopic surgery for Cushing’s disease in the long term.
Castle-Kirszbaum et al., 2022 [17]	52 incidental pituitary adenomas (out of 366 total adenomas resected)	Pituitary macroadenomas	Complete resection achieved in 84.6%	Vision: Among those with visual field deficits (n = 7), vision was improved in 100% (7/7). Endocrine: Two (3.8%) cases of new cortisol deficiency; Three (5.8%) transient DI postoperatively; four (7.7%) postoperative hyponatremia	No perioperative complications reported. No CSF leak cases reported. No mortality reported	All surgical indication subgroups showed a mean improvement in QoL by three months. Age and tumor size did not significantly affect QoL benefit
Nie et al., 2022 [18]	273 patients total; 185 underwent TCS, 88 underwent EES	Craniopharyngiomas (benign epithelial tumors, typically WHO grade I)	Gross Total Resection (GTR) rate: EES: 89.8% TCS: 77.3% (difference statistically significant)	Visual: Among patients with preoperative visual deficits, improvement rates: EES: 74.5% improved, TCS: 56.3% improved (statistically significant difference). Endocrine: Hypopituitarism: EES group 53.4% vs TCS 68.1% (lower in EES, p<0.05); DI: EES 51.1% vs TCS 72.4% (lower in EES, p < 0.05)	CSF leak: EES group 4.5%, TCS group 0% (statistically more frequent in EES). Wound infection: EES 2, TCS 8 (no significant p-value). Meningitis: EES 2, TCS 4 (no significant difference), Hemorrhage: EES 2, TCS 3 (no significant difference), Seizures: EES 1, TCS 4 (no significant difference), Death: No deaths in either group	EES achieved a higher GTR rate than TCS with lower rates of hypopituitarism and DI, better visual improvement rate, and lower recurrence (12.5% EES vs 23.8% TCS). However, EES carries an increased risk of CSF leak compared to TCS; but no mortality difference was seen. Overall, they support that EES is safe, effective, minimally invasive, and in many respects superior to TCS for craniopharyngioma, where anatomically feasible.
Feng et al., 2022 [19]	29 surgeries in 28 patients (i.e., one patient had two operations)	Recurrent craniopharyngiomas	GTR: 16/29 (55.17 %); STR: 11/29 (37.93 %); partial resection: 2/29 (6.90 %)	Out of 22 with preoperative visual impairment, 18 had improvement, three had no change, and one had deterioration; six had normal vision pre- and post-op. Endocrine: Among five patients with normal preoperative pituitary hormone function, only one remained with normal endocrine function postoperatively; the other four developed involvement of one or more hypothalamic-pituitary axes. None of the patients who had preoperative endocrine dysfunction improved. New diabetes insipidus (postoperative): six patients developed DI de novo.	CSF leak: one patient; Bacterial meningitis: one patient (treated with antibiotics + lumbar drain). No serious morbidity or mortality was reported in the cohort	Revision surgery for recurrent craniopharyngiomas is more challenging and carries a higher risk; EETS remains a safe and effective option in appropriately selected cases. They stress that treatment must be individualized, given tumor and patient heterogeneity.
Solari et al., 2023 [20]	84 patients	Craniopharyngiomas, specifically ICs	GTR achieved in 54/84 = 64.28%	Visual: postoperative visual function deteriorated in 5/84 patients (5.95 %) Endocrine: endocrine function deteriorated (new or worsened deficits) in 41/78 patients (52.56 %) Hypothalamic: hypothalamic function deterioration in 14/84 (16.67 %) Recurrence/PFS: 28 patients (33.33 %) had recurrence over follow up (mean follow-up 63.51 months) with five-year PFS ~ 58%.	Cumulative postoperative complication rate: 34.53 %, Most frequent: CSF leak in 14.28 % (≈12 / 84)	EEA offers a direct route to ICs, allows removal even when the tumor extends toward the third ventricle without breaching the diaphragm, and can achieve satisfactory GTR and functional outcomes, but higher surgical complexity and morbidity are expected in tumors with more extensive suprasellar extension and involvement of adjacent neurovascular structures. They note that achieving GTR is associated with better PFS than subtotal resections.
Gong et al., 2022 [21]	73 patients with rrPAs underwent ETS	rrPAs	GTR: 41/73 = 56.1 % GTR + NTR: ~93.2 %	Visual: In the rrPA cohort, vision improved in 9/41 (22.0 %) patients; in the matched non-rrPA cohort, 24/38 (62.5 %) improved. Endocrine/Hypopituitarism/Remission: They observed trends that reoperation in rrPAs had lower rates of recovery of hypopituitarism, lower biochemical remission, and higher rates of new hypopituitarism compared to non-rrPA, but these did not reach statistical significance.	Intraoperative CSF leak: more frequent in rrPA than in non-rrPA (risk increased). No significant difference in overall postoperative complication rates between rrPA vs non-rrPA after propensity matching. Longer postoperative hospital stay in rrPA cohort vs non-rRPA cohort	Knosp grade is an independent predictor of achieving GTR in rrPAs (higher Knosp → lower odds of GTR). Although rrPA patients had higher CSF leak rates and longer hospital stays, there was no significant difference in final GTR rates or overall complication rates when compared to the non-rrPA cohort after matching. They conclude that ETS is a reasonable approach for rrPAs, and that prior surgery or tumor invasion features (Knosp) are more relevant in predicting outcomes than simply recurrence status per se.
Boetto et al., 2022 [22]	53 patients	NFPAs	GTR: achieved in ~70 % of patients. Mean extent of resection overall: ≈ 96 % Predictors: more experience (later cases) positively correlated with higher EOR, and Knosp grade 4 negatively correlated with EOR	Vision improved by 82%	Overall complication rate: 22% (i.e., ~1 in 5 patients). No severe neurological complications reported. The complication rate did not differ significantly between the “first period” (first 30 cases) vs the “second period” (the next 23 cases)	Even during the initial learning phase, NFPA surgery with ETS can be safe and effective with reasonably high resection rates and acceptable complications. The learning curve is evident: surgeon experience (i.e., later cases) correlates with a better EOR. More difficult tumor characteristics (e.g., Knosp grade 4) hamper achieving full resection. The authors advocate for careful patient selection, multidisciplinary support, and intensive training to shorten and smooth the learning curve.
Eguiluz-Melendez et al., 2024 [23]	37 patients with GPAs operated solely via the EEA	GPAs — non-specified whether functional vs non-functional in abstract	GTR in 40.5% of patients	Visual: Favorable visual acuity outcome in 75%; favorable visual field outcome in 82.9%. The abstract does not report explicit endocrine (pituitary hormone) outcomes in its summary	Postoperative CSF leak in 10.8% (as a negative predictor of visual acuity outcome). Vascular injury occurred in 13.5% (associated with intraoperative CSF leak). The abstract mentions morbidity but does not list all complication types (e.g., endocrine, DI)	EEA is a safe and effective technique for GPAs, with acceptable visual preservation, though GTR is challenging. They identify predictors of worse visual acuity outcome: bilateral cavernous sinus invasion (p = 0.018) and postoperative CSF leak (p = 0.036). Intraoperative CSF leak is a predictor of postoperative CSF leak (p = 0.042) and vascular injury (p = 0.048). Radiation therapy is also a predictor for visual field outcome (p = 0.035)
Uzuner et al., 2023 [24]	105 cases (microprolactinomas, < 10 mm)	Microprolactinomas (lactotroph adenomas)	GTR 71%	Endocrine/Remission: On postoperative day 1, remission rate was 85.7 % Long-term remission: 74.3 % over mean follow-up (~74.9 months). The abstract does not mention visual outcomes (since microprolactinomas rarely cause visual compression)	Surgical complication rate: 4.76 % (i.e., about five per 100)	In well-selected patients and when performed by an experienced neurosurgeon, endoscopic transnasal surgery may yield higher cure rates than conventional medical therapy in microprolactinomas patients who had used DAs for more than three years had lower long-term remission rates compared with those with shorter or no DA use (p = 0.01) The authors suggest that surgery should be considered as a viable alternative or even first-line option in selected microprolactinomas, especially for those who are resistant or intolerant of DA therapy.
Oh et al., 2021 [25]	168 patients with NFPAs undergoing ETS	NFPAs	GTR 66%	Vision improved 70%	Postoperative DI occurred in 77/168 = 45.8% of patients. Permanent DI in 10/168 = 6.0%. Median onset of DI: postoperative day 1. Median duration of transient DI: 5 days. In multivariable logistic regression, cephalocaudal tumor diameter had an OR = 2.59 (95% CI 1.05–6.36), p = 0.038 for predicting DI ROC analysis: AUC = 0.68 (95% CI 0.59–0.76), optimal cutoff = 2.7 cm	Among patients undergoing ETS for non-functioning pituitary adenomas, a larger cephalocaudal tumor diameter is an independent predictor of postoperative DI. They propose a cutoff of 2.7 cm as the threshold above which the risk rises.
Simander et al., 2025 [26]	69	Pituitary adenomas	GTR 63%	Visual stable/improved 78%	They define “intraoperative events” and “postoperative complications” (within 3 months). Higher tumor volume was associated with increased risk of intraoperative events. Interestingly, higher age was associated with a decreased risk of postoperative CSF leakage. They examine the role of ISP in relation to complications, but the abstract does not report a statistically significant direct association of ISP with most complications	Tumor size is a risk factor for intraoperative events, and older patients may have a lower risk of CSF leak postoperatively. ISP measurements might help understand or predict complication risk, though the associations are not robustly demonstrated in the abstract. This work builds on their earlier research on ISP in pituitary adenomas (e.g., correlating ISP with tumor volume) to relate pressure to surgical risk.
Vivancos et al., 2025 [27]	117 patients	NFPAs	GTR was achieved (yes/no), and its association was analyzed	Endocrine: Hormonal normalization at 12 months: 13% of patients; Endocrine improvement (≥1 axis) at 12 months: 16.7%; Worsening of hormonal function in ≥1 axis at 12 months: 19.8% younger age associated with hormonal improvement (p = 0.004), Higher preoperative tumor volume (p = 0.015) and absence of GTR (p = 0.049) associated with worsening of ≥1 axis after surgery	CSF leak 4%	Pituitary stalk radiological status (visible vs non-visible) showed no significant association with endocrine outcomes (preoperative or postoperative). Pituitary gland visibility on MRI was correlated: patients whose glands were visible had fewer hormonal axes affected at 12 months (p = 0.011). Larger tumor volume and not achieving GTR were risk factors for endocrine worsening; younger age favored endocrine improvement. The authors suggest that gland visibility on imaging might serve as a prognostic indicator for endocrine preservation or recovery.
Iranmehr et al., 2020 [28]	29 patients	Craniopharyngiomas	GTR: 62% (i.e. ~18/29). “Almost total resection” (>95 % tumor removal) in 86.2%	Visual: After surgery, visual status either improved or remained unchanged in 92.3% of patients. Endocrine / Pituitary function: Pituitary function remained unchanged or worsened in 34.6% of cases postoperatively.Preoperative pituitary dysfunction in 89.7% Preop D: 58.6%; post-op DI: 69.2%	CSF leak: four patients (13.8%), Meningitis: two patients (6.9%), Perioperative mortality: two patients (6.9%), Tumor recurrence: four patients (15.3%) over a mean follow-up of 25 months	EES with the aim of maximal but safe resection is feasible in craniopharyngiomas; they note that while visual improvement and GTR rates are favorable, major concerns remain about CSF leaks, pituitary dysfunction, and infection risk.
Yu et al., 2021 [29]	40 patients	TSMs	GTR: 38/40 = 95.0%; NTR): 2/40 = 5.0%	Visual: Among 39 patients with preoperative visual impairment, 38 (97.4%) improved, and one had no significant change. Endocrine: The paper does not report detailed endocrine (pituitary hormone) outcomes in the abstract or summary	CSF leak: 3/40 = 7.5%, Meningitis (post-CSF leak): 2/40 = 5.0%, Postoperative hyposmia: 8/40 = 20.0% (three had long-term hyposmia). Intraoperative bleeding: one patient (2.5%) had bleeding of an ACA branch → postoperative acute cerebral infarction	The expanded EEEA is a safe and reliable minimally invasive method for TSM removal. Compared to craniotomy, EEEA may yield better visual outcomes and higher rates of GTR, though with the caveat of risk of CSF leak.
Pala et al., 2025 [30]	36 patients	Pituitary adenomas	GTR achieved in 94.4% (34 of 36)	Endocrine/New hypopituitarism: 8.3% (3/36) developed a new hypopituitarism; all new deficits occurred in patients who had microscopic resection. AVP deficiency/DI: 1 patient (2.7%) had AVP deficiency after microscopic resection. QoL: EQ-VAS improved significantly postoperatively (median from 70 → 85, p = 0.003)	No surgical complications or new neurological deficits were reported in the cohort. Intraoperative CSF leak occurred in 25% of patients (but none resulted in postoperative rhinorrhea)	The endoscopic technique might offer benefit in endocrine preservation—i.e., lower incidence of new hypopituitarism and AVP deficiency, since all new deficits occurred in the microscopic group in this sample. Both techniques achieved high GTR rates (94.4%) in experienced hands. QoL improved significantly postoperatively, regardless of surgical technique, though no statistically significant difference in QoL between techniques was observed in this cohort. The study was underpowered (terminated early), and conclusions must be interpreted cautiously. The authors suggest that further, larger randomized trials are needed to confirm the endocrine advantage of endoscopy.
Baussart et al., 2025 [31]	822 consecutive acromegalic patients treated by EES (1998–2022)	Acromegaly (GH-secreting pituitary adenomas)	GTR 72%-85%	Endocrine/Remission: Overall remission rate: 63% Long-term remission by subgroup: Microadenomas (enclosed): 202/230 = 88%, Macroadenomas without obvious cavernous sinus invasion: 316/452 = 70%, Obvious invasive tumors: 3/140 = 2%, New endocrine deficits: New anterior pituitary deficits in 25/822 (3%) New DI: 30/822 (3.6%)	Hematoma: 0.1%, Nerve palsy: 0.1%, CSF leak: 1%, Meningitis: 0.6%, Epistaxis: 1.1%	Endoscopic pituitary surgery is effective and safe for acromegaly when performed in high-volume reference centers. Remission rates are high, especially in microadenomas and non-invasive macroadenomas. Preoperative predictors of persistent hypersecretion include: older age, cavernous sinus invasion, and larger tumor diameter (in multivariate analysis). Recurrence: Among patients with early remission, recurrence of somatotroph hypersecretion occurred in 19/540 (4%), with a mean time to recurrence of ~37.5 months
Kato et al., 2025 [32]	22 pediatric patients underwent 35 EES	Pediatric craniopharyngiomas	GTR 68%-80%	Endocrine (pituitary function): In the group with preoperative preserved function (“Group P”): MPPS was achieved in 18/25 cases (72%). Among those MPPS cases, postoperative preservation of some endocrine function occurred in 14/18 (77.8 %). Infradiaphragmatic tumor locations and intraoperative MPPS were strongly associated with postoperative preservation of some endocrine function (P < 0.001). However, intraoperative MPPS was also significantly associated with risk of tumor recurrence (P = 0.044). They divided patients into “Group P” (some preserved function preop) vs “Group ACL” (already complete endocrine loss preop) for analysis	Tumor recurrence was evaluated in all patients. Intraoperative MPPS preservation was a risk factor for recurrence (P = 0.044). The abstract does not provide a detailed breakdown of surgical complications (e.g., CSF leak, DI, hemorrhage) in its summary	In pediatric craniopharyngiomas treated by EES, postoperative preservation of some endocrine function is feasible especially in infradiaphragmatic tumors and when the pituitary stalk is morphologically preserved However, attempting to preserve the stalk (MPPS) must be balanced against increased risk of tumor recurrence The authors suggest that in ICs, endocrine preservation should be attempted, but the surgeon must be cautious about recurrence risk when preserving the stalk

This series from Kinshasa demonstrates that comprehensive EETS can be implemented safely in a Central African referral network and that outcomes, especially visual, are clinically meaningful despite uneven access to navigation, intraoperative imaging, and standardized vascularized reconstruction. Our cohort was dominated by macroadenomas with smaller proportions of craniopharyngioma and malignant sellar disease, a distribution typical of health systems where delayed presentation and limited MRI access are common [5,7-9,13,14]. Against that backdrop, the core signals of effectiveness in our program, GTR in 56.5% and frequent postoperative visual improvement, track closely with early African experiences and selected LMIC series and fall within the lower bound of results reported by high-volume centers with full adjuncts [13-15,22,33-36].

Extent of resection and disease biology

Our GTR rate (56.5%) mirrors West African series using an anatomy-based technique without routine neuronavigation or universal nasoseptal flaps (Côte d’Ivoire 57%, Senegal 61%) [13,15]. Predictably, cohorts enriched for giant or invasive tumors report lower “complete” resection even with modern optics; for example, Egyptian data in giant adenomas achieved 45% GTR despite contemporary skull-base practice [14]. In contrast, programs with standardized reconstruction, liberal-angled optics, and imaging support report higher GTR (≈66-70%) for nonfunctioning adenomas, even during the learning phase, and ≥90% for selected extra-axial anterior skull-base lesions such as tuberculum sellae meningioma [22,29, 37-39]. These gradients emphasize a constant principle: cavernous sinus invasion and unfavorable consistency, more than surgical philosophy, cap resectability in pituitary disease. Our choice to accept planned subtotal resection in invasive adenomas aligns with propensity-matched data showing that invasion (Knosp grade), rather than “recurrent” status alone, governs achievable EOR and functional yield [21].

Craniopharyngioma behavior in our practice also reflects global experience. Comparative single-institution series and meta-analyses suggest endoscopic endonasal approaches (EEA) increase GTR and visual improvement while reducing hypopituitarism and DI relative to transcranial routes, albeit with a modestly higher CSF-leak risk [18,20,40-42]. Our emphasis on decompression and hypothalamic protection over radiographic radicality is consistent with contemporary stalk-sparing strategies that reserve adjuvant therapy for remnants to protect neuroendocrine function, especially compelling in LMICs, where lifelong hormone replacement imposes heavy social and economic costs [20,43-45].

Functional outcomes

Visual recovery was the most robust clinical benefit in our cohort, consonant with the midline, brain-sparing trajectory of EETS. Improvement rates reported across diverse settings, ≈60%-80% in non-functioning pituitary adenomas (NFPAs) and >70% in craniopharyngioma, provide an external validity frame for our results [13,15,18-20,22]. In selected pathologies, endoscopy’s value extends beyond decompression to quality-of-life (QOL) gains: incidentalomas treated endonasally not only achieved high resection rates but also showed QOL improvement by three months without excess perioperative morbidity [17]. In a randomized comparison, QOL improved after both endoscopic and microscopic surgery, with a trend toward fewer new pituitary deficits after endoscopy, though it was underpowered for definitive superiority [30]. Large acromegaly datasets further support the endocrine efficacy of EETS in expert hands, with long-term remission of 63% overall and >80% in confined microadenomas, and very low rates of new anterior hypopituitarism or AVP deficiency [31].

Endocrine trade-offs remain most consequential in craniopharyngioma. Contemporary pediatric series show that morphological preservation of the stalk increases the probability of retaining some pituitary function but carries a measurable recurrence penalty, underscoring the need to individualize the balance between oncologic control and endocrine life-course outcomes [46]. Our practice to favor safe decompression and accept subtotal resection when hypothalamic planes are hostile accords with those data and with consensus trends [20,32,40,47].

Complications and reconstruction

CSF leakage was our principal technical challenge: transient leaks in 41.5% and persistent leaks in 9.8%. These rates are higher than those reported by high-volume centers (often 5-15% overall) but comparable to LMIC cohorts where vascularized flaps and commercial sealants are not universally available [13-15,22,33,34]. Risk is not purely technical: larger cephalocaudal height and suprasellar extension features common in our case mix predict DI and CSF-related morbidity [25,26]. Even so, layered autologous closure (fat/fascia) and meticulous dead-space control kept infectious complications within acceptable bounds (10.9%), an outcome that echoes other resource-constrained experiences [17,31,38]. As our program matures, universal adoption of multilayer reconstruction with vascularized mucosal flaps where feasible, standardized leak-risk stratification, and protocolized postoperative nasal care should compress our leak rates toward international norms [33,34,46,48].

Two cavernous ICA injuries (4.8%) occurred, rare but widely recognized hazards in invasive adenoma surgery. Similar single-event reports exist in African cohorts, and even large contemporary series report low but non-zero catastrophic vascular risk [13,29,31]. Preoperative mapping of carotid protuberances and opticocarotid relationships, cautious work in the medial cavernous triangle, and early venous bleeding control remain universal safeguards [33,34,49]. Sinonasal morbidity (epistaxis, hyposmia, and paresthesia) in our cohort was self-limited and consistent with prospective data showing favorable sinonasal QOL after endoscopy versus sublabial microscopic exposure when closure is systematic [17,50].

Learning curve, systems, and scalability

Rising performance with experience is well-documented. Even during initial adoption, endoscopic NFPA surgery can deliver high EOR with acceptable morbidity; later cases outperform early ones, and Knosp 4 invasion remains the chief brake on radicality [22]. Our service model, with nearly one-third of operations performed by supervised residents, mirrors residency-embedded experiences that identified predictors of GTR without inflating complications under senior oversight [22]. Yet the most cost-effective performance gains in LMICs often lie outside the operating room: reliable MRI, early endocrine and ophthalmology pathways, ICU protocols for fluid/electrolyte management, and infection control [5,7-9]. Elderly-focused series suggest EETS remains viable in older patients with tailored perioperative goals, an important consideration where ICU beds are scarce [49].

Positioning our results in the global literature

Taken together, our outcomes align with the broad arc of EETS evidence. For pituitary adenomas, our GTR sits in the fifth to sixth decade of percentage points, with visual improvement in a clear majority and endocrine remission achievable in carefully selected functional tumors [13-15,21-23,31,33,34]. Our CSF-leak burden reflects case mix and reconstructive constraints, not a departure from sound technique, and should decrease with standardized multilayer closure and broader availability of vascularized flaps [22,33,34,46,50]. For craniopharyngioma, our conservative stance accords with contemporary data privileging hypothalamic preservation and selective adjuvant therapy, where stakes include the affordability and logistics of lifelong hormone replacement; that trade-off is ethically and clinically salient [18-20,32,40,44,49].

**Figure 9:**
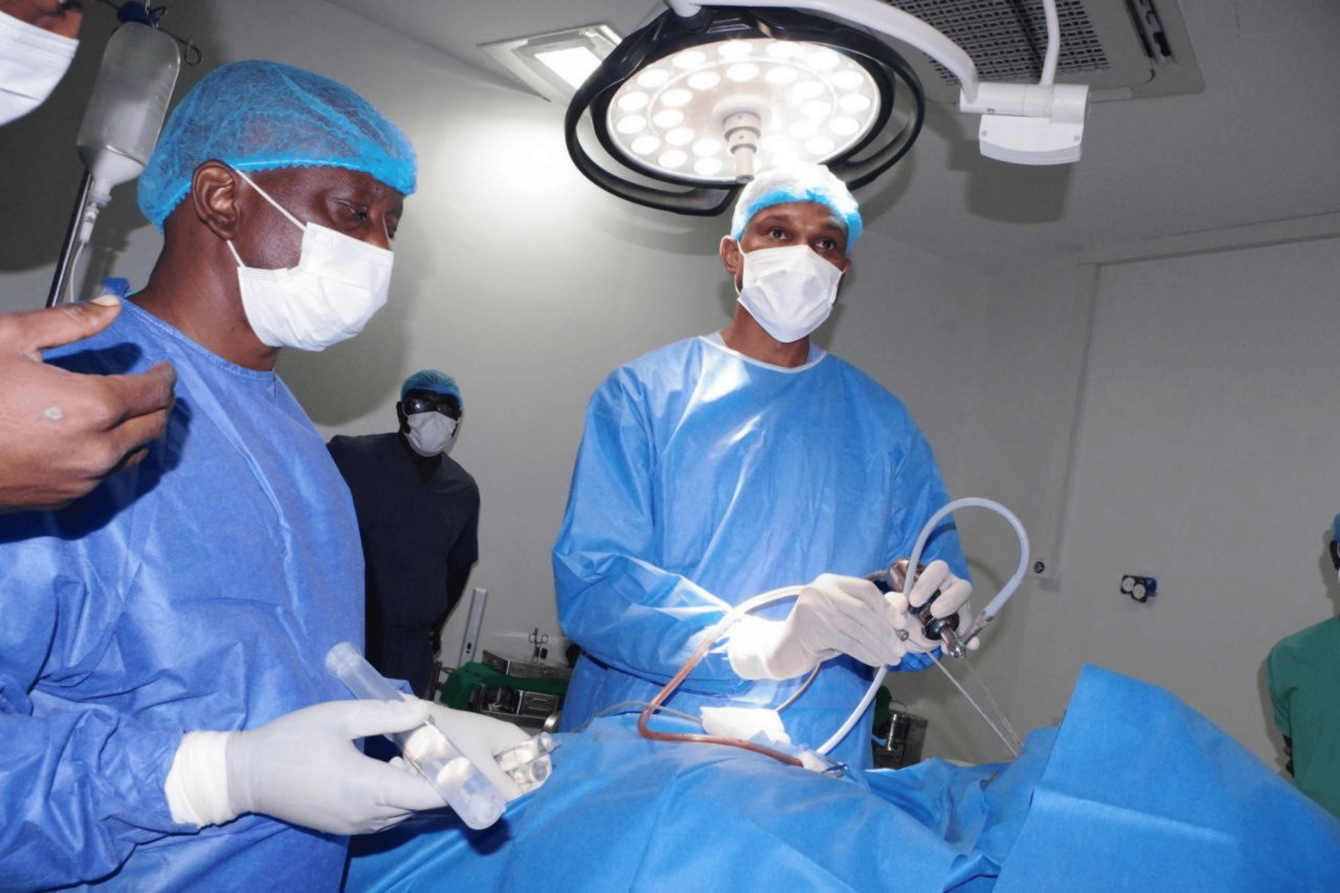
Principal surgeon performing endoscopic endonasal transsphenoidal surgery The principal author is shown operating, assisted by the surgical team. The image demonstrates intraoperative positioning, use of endoscopic instrumentation, and standard sterile setup under the operating light. This reflects the direct involvement of local neurosurgeons in performing and advancing minimally invasive skull base surgery within a Central African institution.

Beyond the clinical outcomes, this experience also highlights the importance of capacity building and local leadership in the adoption of minimally invasive skull base surgery. As shown in Figure 9, the principal author is directly engaged in performing the procedure, reflecting the growing expertise of Central African neurosurgeons in mastering endoscopic techniques. The presence of structured mentorship, hands-on training, and gradual transfer of operative responsibility are crucial steps for ensuring that these procedures become sustainable within local institutions.

Limitations and methodological considerations

This study must be interpreted in light of several important limitations. First, the retrospective design inherently limits causal inference and introduces potential selection bias. Surgical candidacy, choice of approach, and reconstruction technique were influenced by surgeon experience, resource availability, and time period, which cannot be fully standardized post hoc.

Second, the sample size (n = 46) restricts statistical power for subgroup analyses; exploratory associations between tumor morphology, closure technique, and outcomes should therefore be regarded as descriptive rather than confirmatory. Third, operative heterogeneity, including variable access to neuronavigation, angled optics, and vascularized flaps, means the protocol evolved during the study period. While the stepwise anatomy-based method was consistent, detailed intraoperative nuances were not uniformly documented across institutions. Fourth, follow-up duration was incomplete beyond early postoperative evaluation for several patients, precluding robust assessment of long-term endocrine and visual outcomes.

Fifth, missing or partial data on complications (e.g., diabetes insipidus, sinonasal morbidity) limit precision. All results were reported with explicit denominators to mitigate misinterpretation, but the dataset remains subject to reporting bias. Finally, because this represents an early-phase experience from a single referral network in Kinshasa, the results cannot be generalized to all African or global LMIC centers. The outcomes should be viewed as pragmatic indicators of what can be achieved under defined resource constraints rather than as mature institutional benchmarks. Despite these constraints, the study’s transparency detailing complications, mortality, and contextual barriers adds credibility and provides a reproducible framework for similar programs initiating endonasal skull-base surgery in low-resource environments.

## Conclusions

This early Central African experience demonstrates that EETS is technically feasible and can be performed with acceptable short-term safety under defined conditions. The study provides pragmatic evidence that skull-base surgery can be integrated into low-resource neurosurgical programs through structured mentorship, shared equipment, and progressive skill transfer. Nevertheless, the conclusions are bounded by the retrospective, descriptive design, small sample size, and limited follow-up.

The observed complication profile, particularly CSF leakage and infection, indicates that procedural safety and reproducibility are still developing. Therefore, our results should be viewed as foundational data illustrating early implementation, not as mature performance standards. Future prospective, controlled, and multicenter investigations are warranted to determine long-term efficacy, refine reconstruction protocols, and better define predictors of successful outcomes in low- and middle-income settings. Strengthening data registries, perioperative protocols, and training networks across Africa remains the next critical step toward sustainable skull-base oncology capacity.
